# Staphylococcal Enterotoxin A Induces Intestinal Barrier Dysfunction and Activates NLRP3 Inflammasome via NF-κB/MAPK Signaling Pathways in Mice

**DOI:** 10.3390/toxins14010029

**Published:** 2022-01-01

**Authors:** Chunmei Liu, Kunmei Chi, Meng Yang, Na Guo

**Affiliations:** Department of Food Quality and Safety, College of Food Science and Engineering, Jilin University, Changchun 130062, China; liuchunmei0314@163.com (C.L.); chikunmei@163.com (K.C.); mengy20@mails.jlu.edu.cn (M.Y.)

**Keywords:** staphylococcal enterotoxin A, intestinal barrier dysfunction, NLRP3 inflammasome, NF-κB, MAPK

## Abstract

Staphylococcal enterotoxin A (SEA), the toxin protein secreted by *Staphylococcus aureus*, can cause staphylococcal food poisoning outbreaks and seriously threaten global public health. However, little is known about the pathogenesis of SEA in staphylococcal foodborne diseases. In this study, the effect of SEA on intestinal barrier injury and NLRP3 inflammasome activation was investigated by exposing BALB/c mice to SEA with increasing doses and a potential toxic mechanism was elucidated. Our findings suggested that SEA exposure provoked villi injury and suppressed the expression of ZO-1 and occludin proteins, thereby inducing intestinal barrier dysfunction and small intestinal injury in mice. Concurrently, SEA significantly up-regulated the expression of NLRP3 inflammasome-associated proteins and triggered the mitogen-activated protein kinase (MAPK) and nuclear factor kappa-B (NF-κB) signaling pathways in jejunum tissues. Notably, selective inhibitors of MAPKs and NF-κB p65 ameliorated the activation of NLRP3 inflammasome stimulated by SEA, which further indicated that SEA could activate NLRP3 inflammasome through NF-κB/MAPK pathways. In summary, SEA was first confirmed to induce intestinal barrier dysfunction and activate NLRP3 inflammasome via NF-κB/MAPK signaling pathways. These findings will contribute to a more comprehensive understanding of the pathogenesis of SEA and related drug-screening for the treatment and prevention of bacteriotoxin-caused foodborne diseases via targeting specific pathways.

## 1. Introduction

Bacterial toxins are considered to be one of the most common causes of foodborne disease outbreaks worldwide [1]. *Staphylococcus aureus* (*S. aureus*), a common foodborne pathogen, causes foodborne poisoning and toxic shock syndrome by secreting bacterial superantigen toxins (staphylococcal enterotoxins, SEs) [2]. Staphylococcal food poisoning (SFP) caused by the ingestion of pre-formed SE contaminated food is one of the most prevalent bacterial foodborne diseases, and can cause severe food safety, public health and economic problems [3]. The corresponding symptoms mainly include nausea, vomiting, abdominal cramping and diarrhea. Remarkably, SEs are widely distributed in food processing environments, workers’ skin, food production animals, and frequently contaminate raw milk and dairy products, raw meat and meat products, ready-to-eat food, etc. [4,5]. Notably, SEs toxin protein is highly resistant to harsh-sterilization environments during food processing (freezing, drying, heat treatment, acid, alkali), and are even resistant to proteolytic enzymes (including pepsin, trypsin, renin and papain) [4,6]. Besides, previous studies have also found that strong fluorescence intensity (using an anti-SEA antibody) was observed in the jejunum and ileum after oral intake of the SEA protein [2]. Therefore, it can be clearly inferred that the SEA protein can tolerate protease digestion and still maintain biological activity in the intestine after oral intake, further leading to foodborne diseases. At present, more than 20 SEs have been identified [7]. Among them, SEA is the most common enterotoxin involved in global SFP outbreaks, for example, 77.8%, 69.7% and 56.9% of all outbreaks in the United States, France and United Kingdom, respectively [8]. Accordingly, SEA could induce mast cell degranulation and histamine release by binding to the submucosal mast cells in the intestinal tract. The stimulation was transmitted from the gut to the brain through nerves, triggering emetic reaction [9]. Moreover, SEA also exerts strong superantigen activity, which can activate diverse T cells and lead to an inflammatory response even at an extremely low dose [10,11]. As a consequence of its pathogenicity and stability, SEA is considered a serious threat to global public health. However, there are few reports illustrating whether SEA can cause histopathological damage and an inflammatory response in the intestine.

The intestine is not only the main place for nutrient digestion and absorption, but also the first immune barrier against harmful substances, such as pathogenic microorganisms and microbial toxins [12,13]. Therefore, maintaining the function and integrity of the intestinal barrier is essential for human health. The intestinal epithelial barrier is composed of epithelial cells, a mucus layer and intercellular tight junction (TJ) proteins [14]. Tight junctions are a complex of transmembrane proteins, including zonula occludens (Zos), occludin and claudins, and can prevent the translocation of bacterial toxins in the intestinal cavity via linking to the actin cytoskeleton, which is of great significance for maintaining cell bypass and the intestinal barrier [15,16]. Once TJ proteins and the intestinal barrier are damaged, bacterial toxins penetrate the intestinal wall and promote the secretion of pro-inflammatory cytokines, thereby provoking the occurrence of numerous intestinal diseases [14,17].

Furthermore, intestinal epithelial barrier dysfunction is related to the progression of intestinal inflammation. Increasing evidence has indicated that the activation of NLRP3 inflammasome was essential for the pathogenesis of intestinal inflammation and intestinal epithelial barrier dysfunction [18,19]. The NLRP3 inflammasome is a vital part of the innate immune system and is composed of NLRP3, caspase-1 and apoptosis-associated speck-like protein containing CARD (ASC). Following recognition of PAMPs or DAMPs, the activation of NLRP3 inflammasome induces the maturation and release of proinflammatory cytokines, thereby triggering the progression of intestinal inflammation [19,20]. It is well known that the activation of NLRP3 inflammasome is often regulated by multiple intracellular signaling pathways, including nuclear factor kappa-B (NF-κB) and mitogen-activated protein kinase (MAPK) complexes [21]. As the key signaling pathways of various inflammatory diseases, the activation of NF-κB and MAPK promotes the secretion of proinflammatory cytokines, resulting in an inflammatory response [22]. Moreover, as the important signal transduction and transcription factors, NF-κB and MAPK pathways were confirmed to regulate the expression of TJ proteins, which was vital to the actin contraction and to mucosal barrier damage [23,24]. Therefore, NF-κB/MAPK mediated activation of NLRP3 inflammasome is an important mechanism of intestinal barrier dysfunction and inflammatory response.

At present, the clear mechanism of staphylococcal foodborne diseases caused by SEs has not been deeply studied. Previous studies have shown that staphylococcal enterotoxin M (SEM) and staphylococcal enterotoxin B (SEB) contribute to intestinal injury during gastrointestinal infection [25,26]. Further, the researchers found that SEA could translocate across the intestinal mucosal barrier with the help of absorptive epithelial cells through endocytosis [2]. However, as far as we know, the role of SEA on intestinal injury and the underlying toxic mechanism remains unclear. Therefore, the purpose of this study was to investigate the effect of SEA on intestinal barrier dysfunction and NLRP3 inflammasome activation, and to clarify the potential molecular mechanism.

## 2. Results

### 2.1. Preparation of SEA Protein

**Figure 1:**
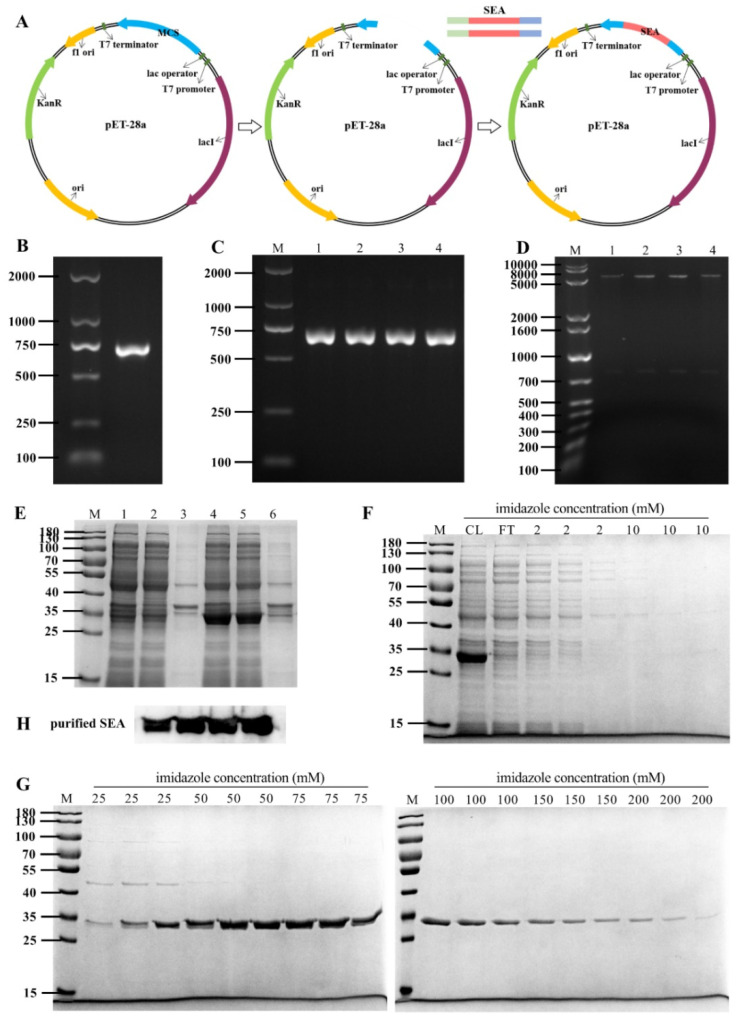
Expression and purification of SEA protein. (**A**) Schematic diagram of constructing pET-28a-SEA expression vector. (**B**) PCR amplification products of *sea* gene. (**C**) Dentification of pET-28a-SEA expression vector by PCR assay (lanes 1–4). (**D**) pET-28a-SEA plasmid was verified by double enzyme digestion using *Bam*H I and *Sal* I (lanes 1–4). (**E**) Sodium dodecyl sulfate polyacrylamide gel electrophoresis (SDS-PAGE) analysis of SEA protein expression induced by isopropyl β-D-thiogalactopyranoside (IPTG). Lanes 1–3: Lysates, supernatants and precipitates of uninduced *E. coli* BL21(DE3) cells. Lanes 4–6: Lysates, supernatants and precipitates of *E. coli* BL21(DE3) cells induced with 0.5 mM IPTG. (**F**–**G**) SDS-PAGE analysis of SEA protein purification by gradient concentration imidazole elution in Ni-NTA affinity chromatography. (**H**) Western blot analysis of purified SEA protein with anti-SEA antibody.

To prepare SEA toxin protein, the full-length SEA toxin coding gene was inserted into the pET-28a expression vector (Figure 1A). As shown in Figure 1B, the *sea* gene was amplified by PCR to obtain a 700 bp amplicon, which was then cloned into a pET-28a plasmid. The recombinant plasmid was transferred into *E. coli* DH5α. Subsequently, the positive clones were screened by PCR and double digestion (Figure 1C,D). These results indicated that the *sea* gene was successfully inserted into the pET-28a vector. Finally, the pET-28a-SEA recombinant plasmid was sequenced, and the nucleotide sequence of the recombinant SEA protein was 99% similar to that reported in GenBank (GenBank accession numbers: M18970, https://www.ncbi.nlm.nih.gov/nuccore/M18970.1/, accessed on 17 October 2021).

Furthermore, the existing form of SEA protein in the expression host *E. coli* BL21 (DE3) strain was analyzed by SDS-PAGE after induction with IPTG. As shown in Figure 1E, compared with the uninduced control group, IPTG (0.5 mM) induced significant expression at 28 °C for 4 h. The expressed SEA protein was identical to the reported protein with molecular weight of about 30 kDa, and the recombinant SEA protein was demonstrated to be soluble expression. The purified SEA toxin protein was obtained by Ni-NTA affinity chromatography and imidazole elution with gradient concentration, and the purity was determined to be greater than 95% (Figure 1F,G). The purified protein was further confirmed as SEA protein by Western blot analysis using an anti-SEA antibody (Figure 1H).

### 2.2. SEA Induced Histological Injury in the Small Intestine of Mice

**Figure 2:**
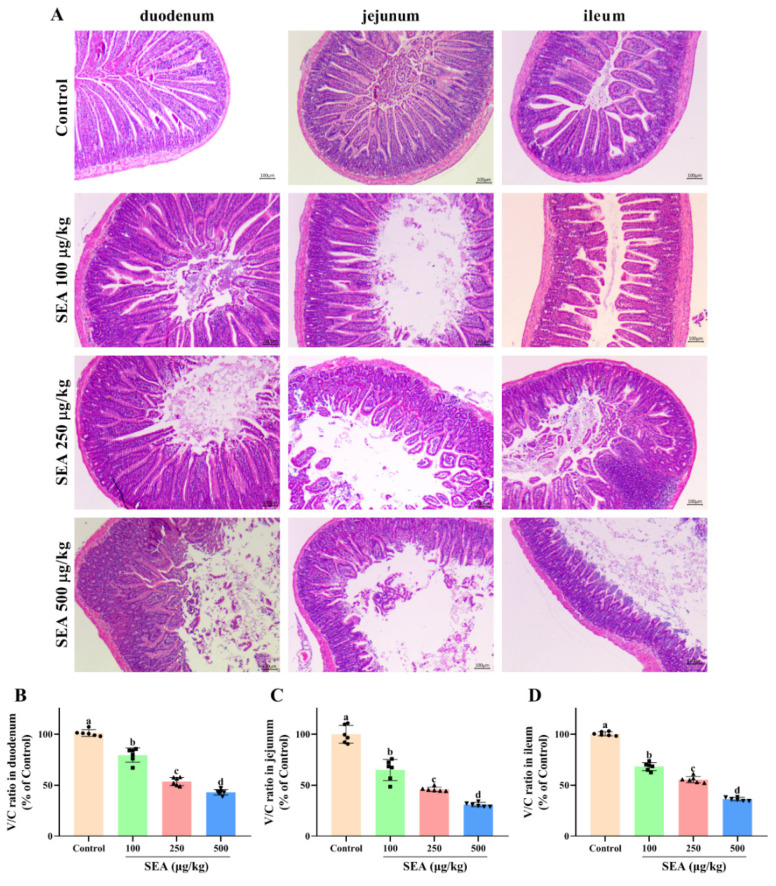
Intestinal injury induced by SEA exposure in mice. (**A**) Representative HE-stained images of the duodenum, jejunum and ileum tissues (100×, scale bar: 100 μm). (**B**) The ratio of villus height to crypt depth (V/C) of duodenum tissues. (**C**) V/C ratio of jejunum tissues. (**D**) V/C ratio of ileum tissues. All data were expressed as mean ± SD (*n* = 6). Different lowercase letters showed significant differences between different groups, *p* < 0.05.

The inflammation and injury of small intestine tissues induced by SEA were evaluated by histopathological analysis using hematoxylin and eosin (HE) staining. As shown in Figure 2A, the small intestine tissues in the control group exhibited normal morphology and well-arranged epithelial cells with normal intestinal villi. In contrast, the small intestines of SEA-treated mice were severely damaged accompanying destruction of the intestinal epithelium, loss of goblet cells, sporadic loss of nuclei staining, obvious inflammatory cell infiltration, villous blunting, distortion of the crypt and overall tissue architecture. Additionally, the injury levels of duodenum, jejunum and ileum tissues were quantified by measuring the ratio of villus height to crypt depth (Figure 2B–D). Compared with the control group, administration of SEA markedly reduced the ratio of villus/crypt (V/C) of duodenum, jejunum and ileum to 43.1%, 30.9% and 36.4%, respectively.

### 2.3. SEA Suppressed the Expression of Tight Junction Proteins of Small Intestine in Mice

**Figure 3:**
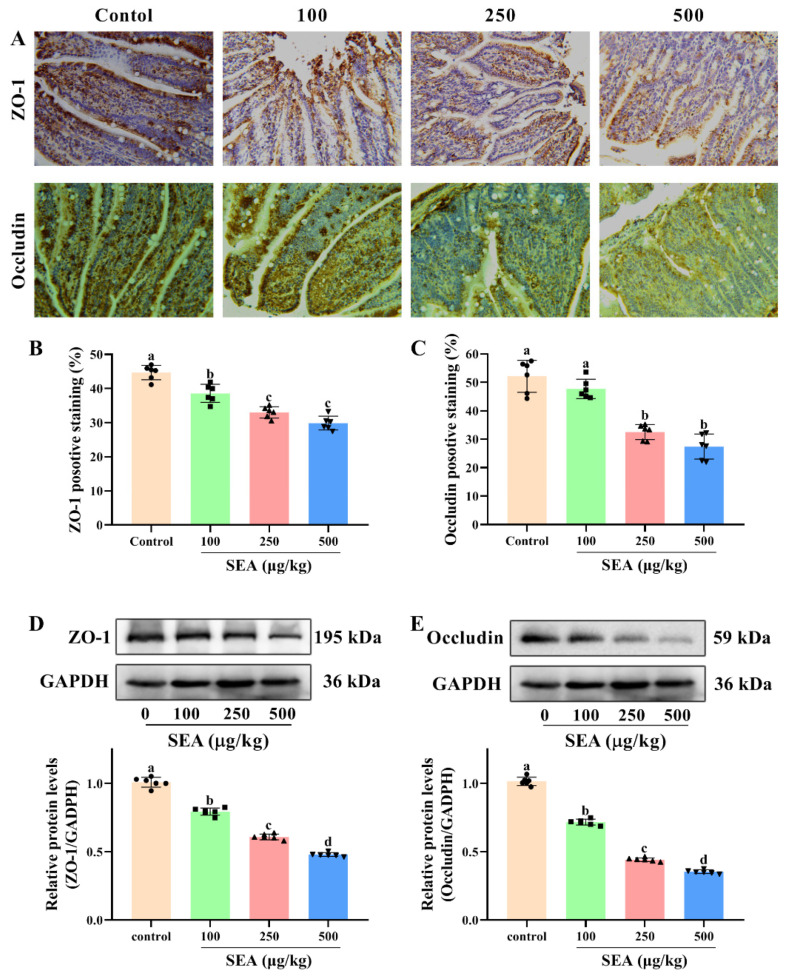
SEA downregulated the expression of tight junction proteins in the jejunum of mice. (**A**) The representative immunohistochemical images of ZO-1 and occludin in the jejunum tissues after SEA administration. (**B**–**C**) The quantitative analysis of IHC results of ZO-1 and occludin. (**D**–**E**) Western blot analysis of ZO-1 and occludin proteins expression in jejunum tissues of SEA-challenged mice. All data were expressed as mean ± SD (*n* = 6). Different lowercase letters showed significant differences between different groups, *p* < 0.05.

The intestinal barrier is responsible for regulating the stable intestinal environment and relieving intestinal inflammation [27]. Intestinal barrier integrity is maintained by tight junction (TJ) proteins. ZO-1 and occludin are essential intestinal epithelial TJ proteins. To determine whether the SEA-mediated damage affected the intestinal barrier function, Western blot and immunohistochemical (IHC) analysis were employed to analyze the expression of ZO-1 and occludin in jejunum tissues. In the IHC experimental results (Figure 3A), brown and light brown sections were considered to be positive expressions. Results showed that there were well-organized and abundant ZO-1 and occludin proteins in the jejunal epithelial cells of the control group. However, SEA stimulation greatly reduced the positive area of ZO-1 and occludin proteins (*p* < 0.05, Figure 3A–C). Similarly, in the results of Western blot, SEA treatment dramatically downregulated the expression of ZO-1 and occludin proteins in a dose-dependent manner (*p* < 0.05, Figure 3D,E, the results of duodenum and ileum were shown in Figures S1 and S2), which showed that the TJ structure was disrupted. Collectively, these results suggested that SEA destroyed the function of the intestinal epithelium and mucosal barrier by reducing the level of TJ proteins, thereby resulting in intestinal injury in mice.

### 2.4. SEA Activated NLRP3 Inflammasome in Jejunum Tissues

**Figure 4:**
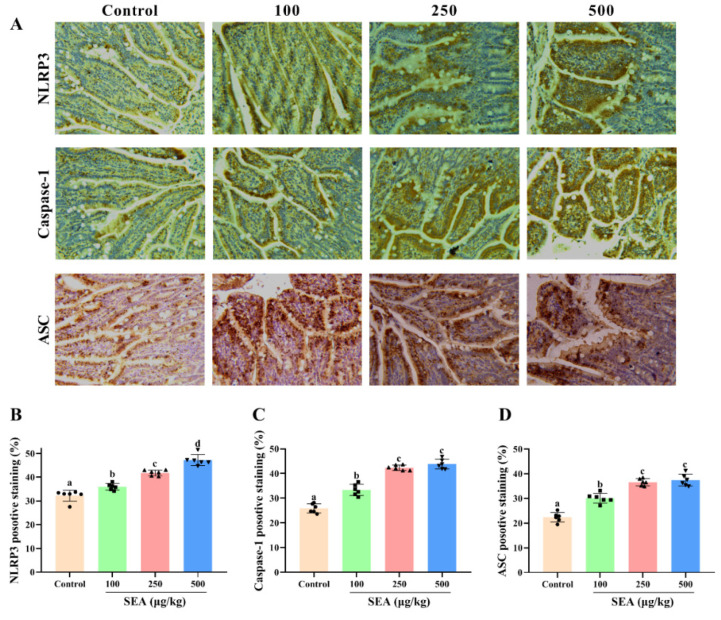
SEA up-regulated the expression of NLRP3 inflammasome related proteins in jejunum tissues of mice. (**A**) The representative immunohistochemical images of NLRP3, caspase-1 and ASC in the jejunum tissues of mice treated by SEA. (**B**–**D**) The quantitative analysis of IHC results of NLRP3, caspase-1 and ASC. All data were expressed as mean ± SD (*n* = 6). Different lowercase letters showed significant differences between different groups, *p* < 0.05.

**Figure 5:**
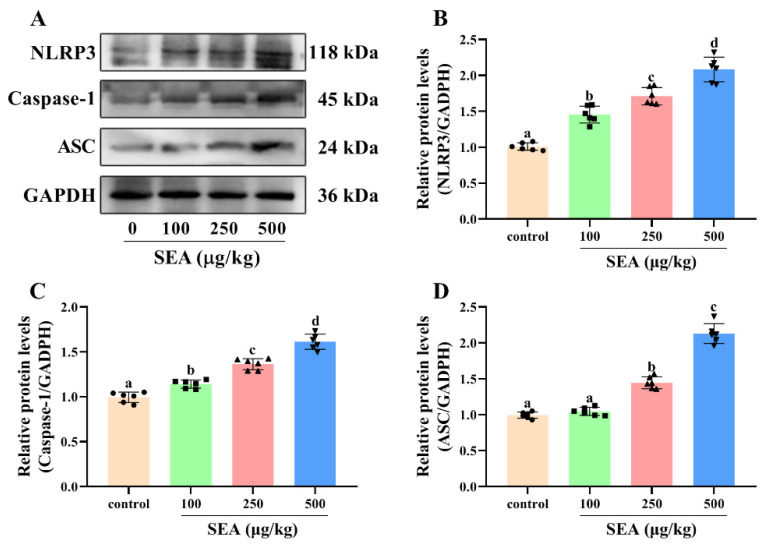
SEA increased the expression levels of NLRP3 inflammasome related proteins in jejunum tissues. (**A**–**D**) Relative protein expression of NLRP3, caspase-1 and ASC in jejunum tissues of SEA-exposed mice. All data were expressed as mean ± SD (*n* = 6). Different lowercase letters showed significant differences between different groups, *p* < 0.05.

**Figure 6:**
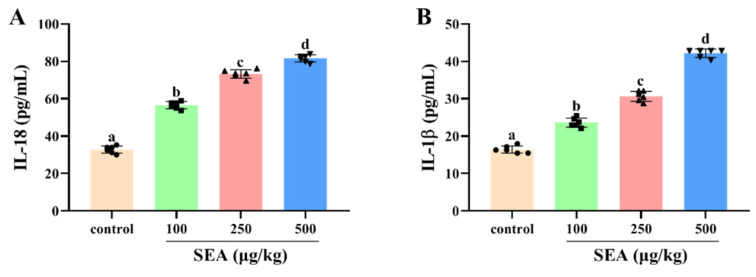
SEA increased the release levels of pro-inflammatory cytokines in mouse serum. The secretion of IL-18 (**A**) and IL-1β (**B**) in serum from mice treated with different stimulants was detected by ELISA. All data were expressed as mean ± SD (*n* = 6). Different lowercase letters showed significant differences between different groups, *p* < 0.05.

Based on the key role of NLRP3 inflammasome in TJ disruption and intestinal injury, Western blot and IHC experiments were performed to determine the effect of SEA toxin protein on the expression of NLRP3 inflammasome related proteins in the jejunum tissues of mice. As shown in the IHC results (Figure 4), the positive expression areas of NLRP3, caspase-1 and ASC in mouse jejunum tissues were gradually elevated with the increase of SEA concentration (*p* < 0.05). Similarly, results of the Western blot analysis also showed that SEA exposure dose-dependently enhanced the expression of NLRP3 inflammasome associated proteins (*p* < 0.05, Figure 5A–D; the results of duodenum and ileum were shown in Figures S1 and S2). Additionally, compared with the control group, SEA exposure substantially increased the levels of pro-inflammatory factors IL-18 and IL-1β in serum (*p* < 0.05, Figure 6). To summarise, these results revealed that SEA toxin protein triggered the activation of NLRP3 inflammasome in mouse jejunum tissues.

### 2.5. SEA Induced the Phosphorylation of NF-κB p65 and Activation of the MAPK Pathway in Jejunum Tissues

**Figure 7:**
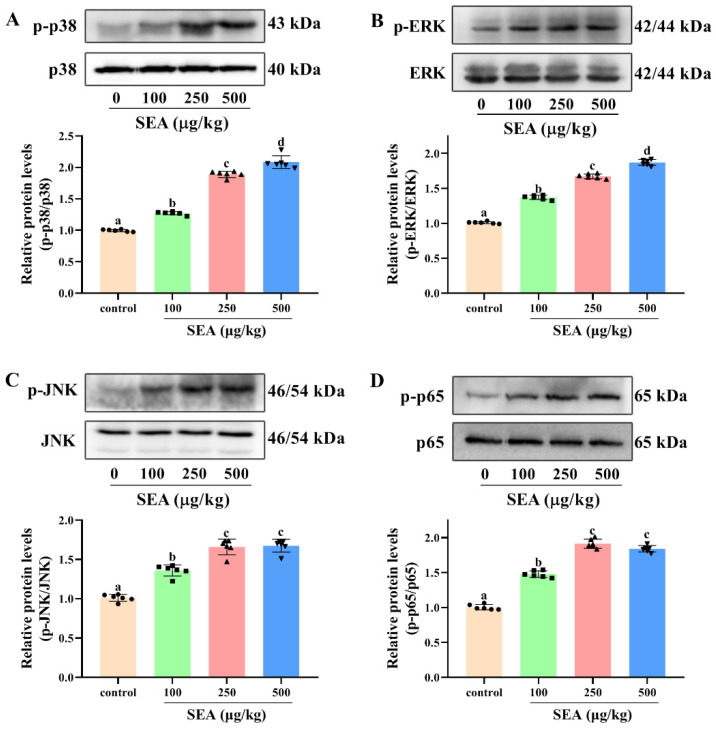
SEA activated NF-κB p65 and MAPK signaling pathways in jejunum tissues of mice. Western blot was used to analyze the phosphorylation level of p38 (**A**), ERK (**B**) and JNK (**C**) in MAPK signaling pathway and p65 (**D**) in NF-κB signaling pathway. All data were expressed as mean ± SD (*n* = 6). Different lowercase letters showed significant differences between different groups, *p* < 0.05.

To further explore the molecular mechanism of NLRP3 inflammasome activation and intestinal barrier destruction, the changes of key proteins in the NF-κB signaling pathway in jejunum tissues of SEA challenged mice were evaluated. The results showed that SEA administration markedly increased the phosphorylation level of NF-κB p65 protein compared with the normal group (Figure 7D, *p* < 0.05). The phosphorylation of NF-κB p65 (Ser311) suggested that SEA stimulation could trigger the NF-κB signaling pathway.

It is well known that the MAPK pathway is also vital for the activation of NLRP3 inflammasome and the occurrence of enteritis. In this study, the changes of key proteins of the MAPK signaling pathway in jejunum tissues stimulated by SEA were measured. Results of the Western blot analysis showed that SEA profoundly promoted the phosphorylation levels of p38 (Thr180/Tyr182), ERK (Thr202/Tyr204) and JNK (Thr183/Tyr185) in the MAPK pathway, and the changes of phosphorylation were dose-dependent (Figure 7A–C, *p* < 0.05). Hence, SEA could activate the MAPK pathway. Combined with the above analysis, SEA might induce the activation of NLRP3 inflammasome and the destruction of the intestinal barrier by regulating NF-κB p65 and MAPK signaling pathways.

### 2.6. SEA Activated NLRP3 Inflammasome via the NF-κB/MAPK Signaling Pathways

**Figure 8:**
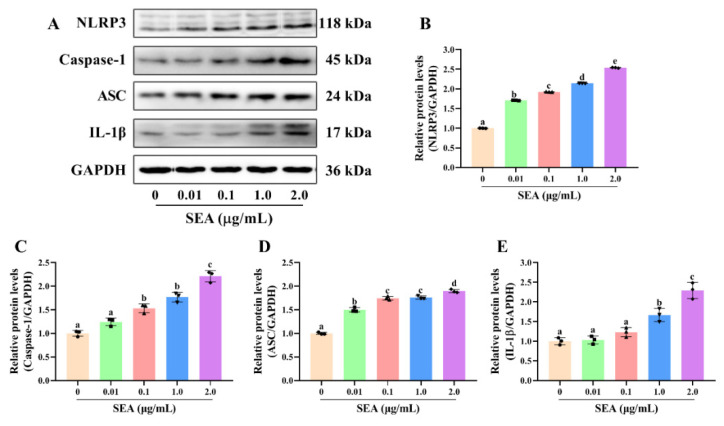
SEA up-regulated the expression of NLRP3 inflammasome related proteins in THP-1 cells. After PMA induction, THP-1 cells were treated with SEA (0.01, 0.1, 1.0 and 2.0 μg/mL) for 24 h. (**A**) Representative immunoblots of the indicated proteins were shown. (**B**–**E**) Relative protein expression of NLRP3, caspase-1, ASC and IL-1β. All data were expressed as mean ± SD. Different lowercase letters showed significant differences between different groups, *p* < 0.05.

**Figure 9:**
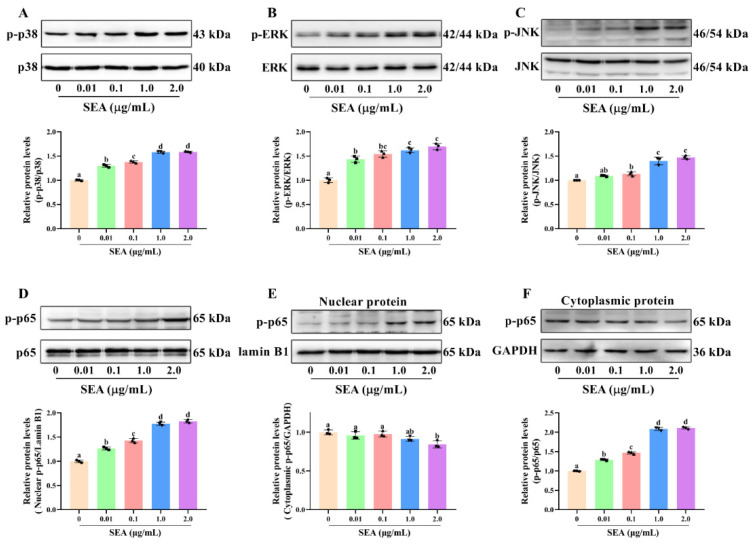
SEA activated NF-κB p65 and MAPK signaling pathways in THP-1 cells. After PMA induction, THP-1 cells were treated with SEA (0.01, 0.1, 1.0 and 2.0 μg/mL) for 24 h. Western blot was used to analyze the phosphorylation level of p38 (**A**), ERK (**B**) and JNK (**C**) in the MAPK signaling pathway and p65 (**D**) in the NF-κB signaling pathway. (**E**,**F**) Western blot analysis was performed to evaluate the protein expression levels of nuclear p-p65 and cytoplasmic p-p65. All data were expressed as mean ± SD. Different lowercase letters showed significant differences between different groups, *p* < 0.05.

As a well-established model to study the mechanism of inflammation, human monocyte cell line THP-1 cells are often used to explore the relationship between signaling pathways [28]. In this study, THP-1 cells were employed to further explore the relationship between NLRP3 inflammasome activation and the NF-κB or MAPK signaling pathways. Consistent with the animal experiments, SEA stimulation activated NLRP3 inflammasome, MAPK and NF-κB p65 signaling pathways in THP-1 cells (Figure 8 and Figure 9A–D). Additionally, SEA toxin protein dose-dependently promoted the translocation of the p65 subunit from the cytoplasm to the nucleus (Figure 9E,F), which further explained the activation of NF-κB pathway by SEA.

**Figure 10:**
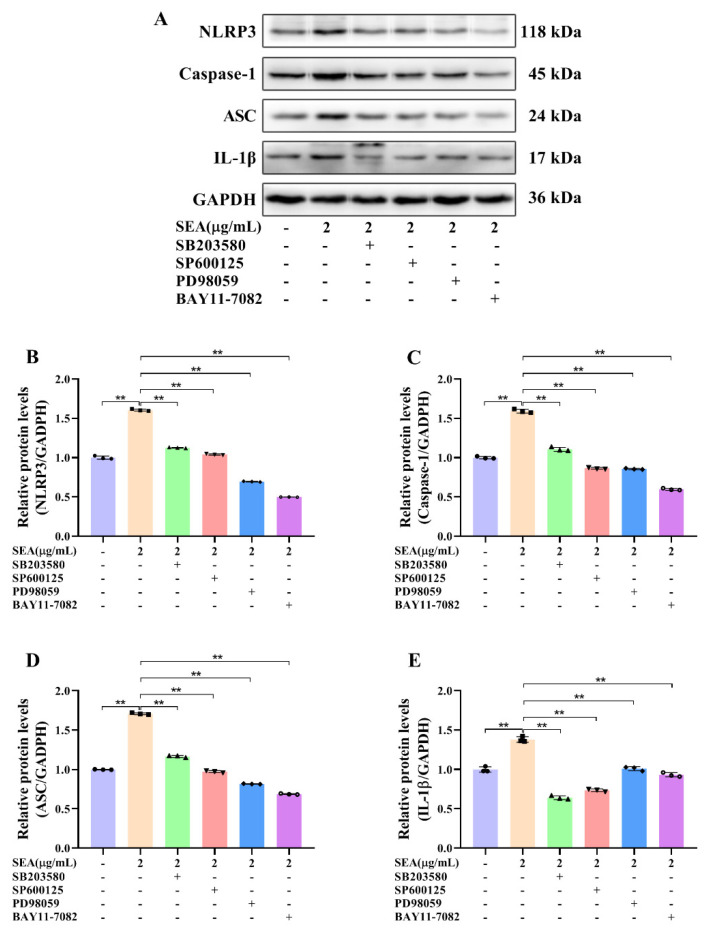
SEA up-regulated the expression of NLRP3 inflammasome related proteins by triggering the NF-κB and MAPK signaling pathways in THP-1 cells. After pretreatment with SB203580 (p38 inhibitor), SP600125 (JNK inhibitor), PD98059 (ERK inhibitor) and BAY11-7082 (NF-κB p65 inhibitor) for 2 h, THP-1 cells were treated with SEA (0.01, 0.1, 1.0 and 2.0 μg/mL) for 24 h. (**A**) Representative immunoblots of the indicated proteins were shown. (**B**–**E**) Relative protein expression of NLRP3, caspase-1, ASC and IL-1β. Data are presented as the mean ± SD. ** *p* < 0.01.

To confirm whether the NF-κB p65 and MAPK signaling pathways participated in the activation of NLRP3 inflammasome triggered by SEA, pretreatment with SB203580 (10 mM), SP600125 (10 mM), PD98059 (10 mM) and BAY11-7082 (10 mM) was performed to inhibit the protein expression levels of p-p38, p-JNK, p-ERK and p-p65, respectively. THP-1 cells were pretreated with inhibitors for 2 h before exposure to SEA, and then the expression levels of NLRP3 inflammasome associated proteins were detected. As shown in Figure 10, inhibitor pretreatment significantly suppressed the expression of NLRP3, caspase-1 and ASC protein up-regulated by SEA in THP-1 cells (*p* < 0.01). Therefore, these analyses demonstrated that SEA could activate NLRP3 inflammasome, at least partially by triggering the NF-κB and MAPK signaling pathways.

## 3. Discussion

**Figure 11:**
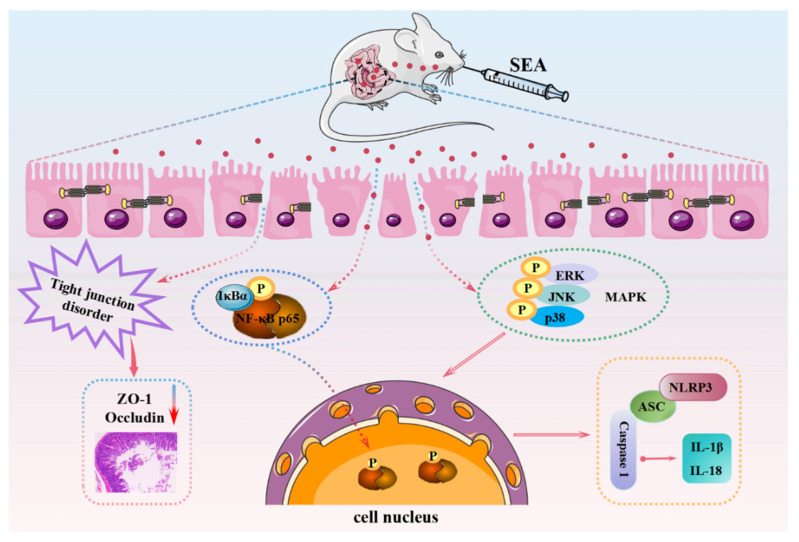
Schematic diagram of the mechanism of SEA inducing intestinal barrier dysfunction and activating NLRP3 inflammasome.

As a consequence of pathogenicity and stability, the health risks associated with enterotoxin exposure have become a global public health concern. However, the clear mechanism of SEA-caused staphylococcal foodborne diseases and the harm of SEA to the intestine has not been fully reported or elucidated. Here, we investigated the effects of SEA on intestinal barrier dysfunction and NLRP3 inflammasome activation and clarified the underlying molecular mechanisms (Figure 11).

In the current study, histopathological analysis showed significant damage to the intestinal tissue architecture with obvious inflammatory cell infiltration, villous blunting, distortion of the crypt and destruction of the intestinal epithelium in SEA-challenged mice. Notably, the height of intestinal villi can not only increase the absorption and utilization of nutrients, but also protect the body from the infection of pathogens and affect the intestinal barrier and immune function [29]. Previous studies have observed strong fluorescence intensity in the jejunum and ileum of the gastrointestinal tract of SEA gavage monkeys by using an anti-SEA antibody [2,9]. In our study, the consistent injury of jejunum tissues by SEA gavage was the most serious, followed by ileum and duodenum tissues. Therefore, SEA caused significant damage to mouse small intestine, especially jejunum tissues, which might affect the intestinal barrier and immune function.

Accumulated evidence has indicated that the intestinal barrier could prevent intestinal mucosal injury by blocking exogenous substances, and has played an essential role in maintaining intestinal health and host immune regulation [30,31]. Tight junction (TJ) proteins, the key part of the intestinal barrier, form a physical barrier to strengthen the protective function of the intestine and play an indispensable role in maintaining intestinal barrier function [32]. The changes of TJ proteins (e.g., ZO-1 and occludin) are able to cause intestinal barrier dysfunction and affect intestinal health [33]. In this study, Western blot and IHC results showed that SEA dose-dependently downregulated the expression of ZO-1 and occludin proteins in mouse jejunum tissues. These results indicated that SEA exposure induced intestinal-barrier dysfunction in mice, which might lead to harmful substances entering the immune system and trigger an inflammatory response. Therefore, the development of compounds to restore intestinal barrier function may be a promising strategy for the prevention and treatment of SEA-caused foodborne diseases [16,24,29].

As a member of the innate immune receptor and NOD-like receptor (NLR) family, NLRP3 inflammasome participates in the cellular inflammatory response and various inflammatory diseases, and exerts a significant impact on intestinal barrier function and infectious enteritis [34,35,36]. NLRP3 recruits ASC and caspase-1 to promote the activation of caspase-1, thus resulting in the maturation and secretion of proinflammatory cytokines and triggering the inflammatory response [32,37]. Previous studies have found that staphylococcal toxins (e.g., α-hemolysin and toxic shock syndrome toxin), could activate NLRP3 inflammasome and cause bacterial toxin infectious diseases, such as pneumonia [38,39]. Similarly, our Western blot and IHC results found that the expression of NLRP3, caspase-1 and ASC proteins was increased in SEA stimulated mouse jejunum tissues. Additionally, SEA also increased the secretion of pro-inflammatory cytokines (IL-18 and IL-1β) in mouse serum. The results strongly supported the idea that SEA toxin protein activated NLRP3 inflammasome in jejunum tissues, which induced an inflammatory response and intestinal injury.

The NF-κB and MAPK signaling pathways are well known to be involved in inflammation and immune regulation [40]. NF-κB is a nuclear transcription factor in cells and regulates the inflammatory response by modulating the transcription of inflammatory genes [18]. In an inflammatory environment, NF-κB p65 is phosphorylated and translocated into the nucleus, thereby promoting the transcription of inflammatory markers and inducing irreversible inflammatory injury, which is the important mechanism for regulating inflammatory diseases [31,32,41]. Additionally, the NF-κB pathway is also a crucial factor responsible for the disruption of the intestinal barrier and activation of NLRP3 inflammasome [36]. In addition to NF-κB, MAPK signaling is also reported to regulate the production of inflammatory factors. MAPK is a family of threonine/serine protein kinases. The activation of the MAPK pathway involves the phosphorylation of JNK, ERK and p38, which regulates the expression of inflammatory genes [31,42]. Importantly, the MAPK signaling pathway has been proved to regulate intestinal TJ proteins [24]. Increasing evidence has indicated that the NF-κB and MAPK signaling pathways have played a crucial role in the pathogenesis of intestinal inflammation and NLRP3 inflammasome activation. Furthermore, it was reported that SEA could activate the NF-κB pathway in human PBMC cells to induce fever and induced MUC5B expression in human airway epithelial cells through phosphorylation of ERK and p38 [43,44]. Therefore, the effect of SEA on the activation of the NF-κB and MAPK pathways was investigated to further explore the molecular mechanism of SEA-induced intestinal barrier dysfunction and NLRP3 inflammasome activation. Our results showed that SEA dramatically enhanced the phosphorylation of p38 (Thr180/Tyr182), JNK (Thr183/Tyr185), ERK (Thr202/Tyr204), and NF-κB p65 (Ser311) in jejunum tissues and THP-1 cells, and induced the nuclear translocation of NF-κB p65 in THP-1 cells. Hence, we speculated that the activation of the MAPK and NF-κB signaling pathways may be involved in the SEA-induced inflammatory response and the activation of NLRP3 inflammasome.

Moreover, to further clarify whether SEA activated NLRP3 inflammasome by triggering the NF-κB and MAPK signaling pathways, THP-1 cells were pretreated with the selective inhibitors of the NF-κB and MAPK pathways before SEA stimulation. As expected, the expression of NLRP3 inflammasome associated proteins up-regulated by SEA stimulation was dramatically attenuated by the selective inhibitors of the NF-κB and MAPK pathways in THP-1 cells. Therefore, it could be clearly revealed that SEA toxin protein induced the activation of NLRP3 inflammasome via the NF-κB and MAPK signaling pathways. More importantly, NF-κB/MAPK-mediated activation of NLRP3 inflammasome may be a target for adjuvant treatment of SEA toxin protein infection in the future.

## 4. Conclusions

In conclusion, our study demonstrated that SEA exposure could cause villi injury and inhibit the expression of TJ proteins, thereby inducing intestinal barrier dysfunction and small intestinal injury in mice. Meanwhile, SEA was first confirmed to activate the NLRP3 inflammasome in the small intestine via the NF-κB/MAPK signaling pathways. Taken together, this study contributes to a more comprehensive understanding of SEA-induced pathogenesis by targeting intestinal barrier dysfunction and the immune inflammatory response, and provides novel insights into the screening of natural bioactive compounds for the treatment and prevention of bacteriotoxin-caused foodborne diseases via targeting specific pathways.

## 5. Materials and Methods

### 5.1. Materials

T4 DNA ligase and *BamH* I and *Sal* I restriction endonuclease were obtained from New England Biolabs Co., LTD. (Beijing, China). Isopropyl β-D-thiogalactopyranoside (IPTG) and Ni-NTA affinity chromatography resin (His-tag purification resin) were bought from Beyotime (Shanghai, China). Inhibitors of c-Jun N-terminal kinase (JNK) (SP600125), p38 (SB203580), extracellular signal-regulated kinases (ERK) (PD98059) and p65 (BAY11-7082) were purchased from MCE (Shanghai, China).

### 5.2. Expression and Purification of SEA

Preparation of SEA toxin protein was completed by *Escherichia coli* expression system and Ni-NTA affinity chromatography [45,46]. In short, the *sea* gene was amplified by polymerase chain reaction (PCR) from *S. aureus*. The primer sequence was: 5-CTC**GGATCC**AGCGAGAAAAGCGAAGA-3 (Forward) and 5-CGC**GTCGAC**CTAACTTGTATATAAATAT-3 (Reverse) (inserting *BamH* I and *Sal* I restriction sites). PCR product and pET-28a plasmid were digested with *BamH* I and *Sal* I, and ligated using T4 DNA ligase at 4 °C overnight. The cloned vector was named pET-28a-SEA and was transferred into *E. coli* DH5α competent cells. The pET-28a-SEA vector was validated by PCR and double digestion. The nucleotide sequence of *sea* gene was also confirmed by Sangon Biotech (Shanghai, China).

Subsequently, the pET-28a-SEA recombinant vector was transferred into *E. coli* BL21 (DE3) cells. And the *E. coli* BL21 (DE3) strain with the pET-28a-SEA vector was induced by IPTG (0.5 mM) for 4 h at 28 °C. The bacteria were resuspended in non-denatured lysate, and the supernatant was collected after ultrasonic breaking. And then, the supernatant containing the recombinant SEA protein was further purified with Ni-NTA affinity chromatography resin, where the SEA protein was eluted with gradient concentration of imidazole (three times per concentration). The purified SEA protein was confirmed by SDS-PAGE and Western blot analysis. Ultimately, SEA protein was dialyzed into 1 mM phosphate buffer (PBS, pH7.4) at 4 °C and then lyophilized in a freeze dryer.

### 5.3. Animals and Experimental Design

Twenty-four male BALB/c mice (6–8 weeks old, 18–20 g) were obtained from the Laboratory Animal Center of Jilin University (Changchun, China). All mice were housed in an air-conditioned room with 12 h light/dark cycle and constant temperature of 24 ± 1 °C, and provided water and food *ad libitum* for 7 days to adapt to the environment prior to experiment. All animal experiments were conducted in accordance with the guidelines of the Animal Care and Use Committee of Jilin University (Approval Number: SY202106013).

After 7 days of acclimatization, the mice were randomly divided into 4 groups (*n* = 6/group): Control group (0 µg/kg SEA), low dose SEA group (100 µg/kg), medium dose SEA group (250 µg/kg), high dose SEA group (500 µg/kg). Mice of the experimental group were fed with different concentrations of SEA in PBS by oral administration. Mice of the control group were administrated with PBS. After 24 h, all mice were euthanized. Then, the serum as well as duodenum, jejunum and ileum tissues were collected for further analysis.

### 5.4. Histopathological Analysis

Histological evaluation of the small intestine was performed according to a previous study [47]. Briefly, small intestinal samples of mice were fixed with 10% neutral formalin and embedded in paraffin. Subsequently, paraffin sections of 4 μm thick were stained with hematoxylin eosin (HE) (Beyotime, Shanghai, China) and were observed under a light microscope (BDS400, OPTEC, Chongqing, China). Villus height and crypt depth of each tissue section were measured by Image J 1.8.0 software (National Institutes of Health, Bethesda, MD, USA). Then, the ratio of villus height to crypt depth (V/C) was calculated to evaluate intestinal injury.

### 5.5. Immunohistochemical (IHC) Analysis

Immunohistochemistry was completed according to the manufacturer’s protocol (Boster, Wuhan, China). Briefly, paraffin sections of jejunum tissues were deparaffinized and rehydrated. Subsequently, the sections were incubated for 10 min with 3% H_2_O_2_, blocked with 5% bovine serum albumin (BSA) for 1 h, and then incubated at 4 °C overnight with primary antibodies against ZO-1 (1:100, Affinity), occludin (1:200, Proteintech), NLRP3 (1:50, Affinity), caspase-1 (1:200, Proteintech) and ASC (1:50, Santa). After that, the tissue sections were incubated with HRP-conjugated secondary antibodies. Finally, signals were developed with diaminobenzidine as a chromogen, counterstained with hematoxylin and then detected by the light microscope (BDS400, OPTEC, Chongqing, China). Integrated optical density (IOD) values were quantified by Image-Pro Plus 6.0 (Media Cybernetics, Bethesda, MD, USA).

### 5.6. Western Blot Analysis

The total proteins of the intestinal tissues and cells were extracted with a RIPA lysis buffer, while the nuclear and cytoplasmic proteins were separated with a Nuclear and Cytosol Protein Extraction Kit (Beyotime, Shanghai, China). Subsequently, the protein concentration was quantitated by BCA Protein Assay Kit (Beyotime). The equal amounts of protein samples were separated by 10% SDS-PAGE gels, and then transferred onto PVDF membranes (Millipore, Bedford, MA, USA). After being blocked with 5% skimmed milk at room temperature for 2 h, PVDF membranes were incubated overnight at 4 °C with primary antibodies (Supplementary Table S1), followed by incubation with secondary antibodies. The protein blots were detected by enhanced chemiluminescence (ECL). The relative protein expressions were normalized to the expressions of GAPDH (cytoplasmic protein and total protein) and Lamin B1 (nuclear protein).

### 5.7. ELISA Assay

The content of IL-18 and IL-1β in the collected serum was detected using the corresponding ELISA kits, following the manufacturer’s protocol (Jianglai Biotech, Shanghai, China).

### 5.8. Cell Culture

Human monocyte cell line THP-1 is a widely used model to study inflammatory mechanisms, cellular signal pathways and monocytes/macrophage functions [28]. THP-1 cells were obtained from the Chinese Academy of Sciences (Shanghai, China) and cultured in RPMI 1640 (Gibco, Thermo Fisher Scientific Inc., Waltham, MA, USA) supplemented with 10% FBS (Gibco, Thermo Fisher Scientific Inc., Waltham, MA, USA) at 37 °C with 5% CO_2_. To induce THP-1 cells to differentiate into macrophages (THP-Ms), the cells were seeded in six-well plates (1 × 10^6^ cells/well) and treated with 100 ng/mL Phorbol-12-myristate-13-acetate (PMA) for 48 h. After that, THP-M cells were stimulated with gradually increasing concentrations of SEA toxin protein (0, 0.01, 0.1, 1.0, 2.0 µg/mL) for 24 h in the presence or absence of pretreatment of SB203580 (p38 inhibitor), SP600125 (JNK inhibitor), PD98059 (ERK inhibitor) and BAY11-7082 (NF-κB p65 inhibitor) for 2 h. Subsequently, cell proteins were extracted to explore the changes of related protein expression through Western blot analysis.

### 5.9. Statistical Analysis

Statistical analysis was performed using GraphPad Prism 8.0 (GraphPad Software, San Diego, CA, USA). The significant differences between groups were analyzed by a one-way ANOVA test followed by Tukey’s post-hoc test, and differences with *p* < 0.05 were considered to be statistically significant. Data were presented as the mean ± standard deviation (SD).

## Data Availability

Data will be provided on request.

## Supplementary Materials

The following are available online at https://www.mdpi.com/article/10.3390/toxins14010029/s1, Table S1. The primary antibodies used in this study; Figure S1. Western blot results of duodenum tissues of SEA-exposed mice. (A) Relative protein expression of ZO-1 and occludin. (B) Relative protein expression of NLRP3, caspase-1 and ASC. All data were expressed as mean ± SD (*n* = 3). Different lowercase letters showed significant differences between different groups, *p* < 0.05; Figure S2. Western blot results of ileum tissues of SEA-exposed mice. (A) Relative protein expression of ZO-1 and Occludin. (B) Relative protein expression of NLRP3, Caspase-1 and ASC. All data were expressed as mean ± SD (*n* = 6). Different lowercase letters showed significant difference between different groups, *p* < 0.05.

## References

[1] Grispoldi L., Karama M., Armani A., Hadjicharalambous C., Cenci-Goga B.T. (2021). *Staphylococcus aureus* enterotoxin in food of animal origin and staphylococcal food poisoning risk assessment from farm to table. Ital. J. Anim. Sci..

[2] Hirose S., Ono H.K., Omoe K., Hu D.L., Asano K., Yamamoto Y., Nakane A. (2016). Goblet cells are involved in translocation of staphylococcal enterotoxin A in the intestinal tissue of house musk shrew (*Suncus murinus*). J. Appl. Microbiol..

[3] Kadariya J., Smith T.C., Thapaliya D. (2014). *Staphylococcus aureus* and staphylococcal food-borne disease: An ongoing challenge in public health. Biomed Res. Int..

[4] Hennekinne J.A., De Buyser M.L., Dragacci S. (2012). *Staphylococcus aureus* and its food poisoning toxins: Characterization and outbreak investigation. Fems. Microbiol. Rev..

[5] Bencardino D., Amagliani G., Brandi G. (2021). Carriage of *Staphylococcus aureus* among food handlers: An ongoing challenge in public health. Food Control.

[6] Le Loir Y., Baron F., Gautier M. (2003). *Staphylococcus aureus* and food poisoning. Genet. Mol. Res. GMR.

[7] Tarisse C.F., Goulard-Huet C., Nia Y., Devilliers K., Marce D., Dambrune C., Lefebvre D., Hennekinne J.A., Simon S. (2021). Highly sensitive and specific detection of staphylococcal enterotoxins SEA, SEG, SEH, and SEI by immunoassay. Toxins.

[8] Argudin M.A., Mendoza M.C., Rodicio M.R. (2010). Food poisoning and *Staphylococcus aureus* enterotoxins. Toxins.

[9] Ono H.K., Hirose S., Narita K., Sugiyama M., Asano K., Hu D.L., Nakane A. (2019). Histamine release from intestinal mast cells induced by staphylococcal enterotoxin A (SEA) evokes vomiting reflex in common marmoset. PLoS Pathog..

[10] Fisher E.L., Otto M., Cheung G.Y.C. (2018). Basis of virulence in enterotoxin-mediated staphylococcal food poisoning. Front. Microbiol..

[11] Shimamura Y., Noaki R., Kurokawa A., Utsumi M., Hirai C., Kan T., Masuda S. (2021). Effect of (−)-epigallocatechin gallate on activation of JAK/STAT signaling pathway by staphylococcal enterotoxin A. Toxins.

[12] Mowat A.M., Agace W.W. (2014). Regional specialization within the intestinal immune system. Nat. Rev. Immunol..

[13] Yue D., Wang Z., Yang Y., Hu Z., Luo G., Wang F. (2021). EZH2 inhibitor GSK343 inhibits sepsis-induced intestinal disorders. Exp. Ther. Med..

[14] Mahmoud T.N., El-Maadawy W.H., Kandil Z.A., Khalil H., El-Fiky N.M., El Alfy T. (2021). *Canna x generalis* L.H. Bailey rhizome extract ameliorates dextran sulfate sodium-induced colitis via modulating intestinal mucosal dysfunction, oxidative stress, inflammation, and TLR4/ NF-B and NLRP3 inflammasome pathways. J. Ethnopharmacol..

[15] Serreli G., Melis M.P., Zodio S., Naitza M.R., Casula E., Penalver P., Lucas R., Loi R., Morales J.C., Deiana M. (2020). Altered paracellular permeability in intestinal cell monolayer challenged with lipopolysaccharide: Modulatory effects of pterostilbene metabolites. Food Chem. Toxicol..

[16] Fan J., Li B.R., Zhang Q., Zhao X.H., Wang L. (2021). Pretreatment of IEC-6 cells with quercetin and myricetin resists the indomethacin-induced barrier dysfunction via attenuating the calcium-mediated JNK/Src activation. Food Chem. Toxicol..

[17] Kim T.I. (2015). The role of barrier dysfunction and change of claudin expression in inflammatory bowel disease. Gut Liver.

[18] Zhong J., Yu R., Zhou Q., Liu P., Liu Z., Bian Y. (2021). Naringenin prevents TNF-alpha-induced gut-vascular barrier disruption associated with inhibiting the NF-kappaB-mediated MLCK/p-MLC and NLRP3 pathways. Food Funct..

[19] Tian M., Ma P., Zhang Y., Mi Y., Fan D.D. (2020). Ginsenoside Rk3 alleviated DSS-induced ulcerative colitis by protecting colon barrier and inhibiting NLRP3 inflammasome pathway. Int. Immunopharmacol..

[20] Lee P.Y., Liu C.C., Wang S.C., Chen K.Y., Lin T.C., Liu P.L., Chiu C.C., Chen I.C., Lai Y.H., Cheng W.C. (2021). Mycotoxin zearalenone attenuates innate immune responses and suppresses NLRP3 inflammasome activation in LPS-activated macrophages. Toxins.

[21] Zhao W.M., Ma L., Cai C., Gong X.H. (2019). Caffeine inhibits NLRP3 inflammasome activation by suppressing MAPK/NF-kappa B and A2aR signaling in LPS-induced THP-1 macrophages. Int. J. Biol. Sci..

[22] Hu P., Zhao F., Wang J., Zhu W. (2020). Lactoferrin attenuates lipopolysaccharide-stimulated inflammatory responses and barrier impairment through the modulation of NF-kappaB/MAPK/Nrf2 pathways in IPEC-J2 cells. Food Funct..

[23] Yang G., Bibi S., Du M., Suzuki T., Zhu M.-J. (2017). Regulation of the intestinal tight junction by natural polyphenols: A mechanistic perspective. Crit. Rev. Food Sci. Nutr..

[24] Yuan J., Che S., Ruan Z., Song L., Tang R., Zhang L. (2021). Regulatory effects of flavonoids luteolin on BDE-209-induced intestinal epithelial barrier damage in Caco-2 cell monolayer model. Food Chem. Toxicol..

[25] Zhao Y., Tang J. (2018). Staphylococcal enterotoxin M causes intestine dysfunction via activating inflammation. J. Food Saf..

[26] Larcombe S., Jiang J.H., Hutton M.L., Abud H.E., Peleg A.Y., Lyras D. (2020). A mouse model of *Staphylococcus aureus* small intestinal infection. J. Med. Microbiol..

[27] Cui Y.J., Okyere S.K., Gao P., Wen J., Cao S.Z., Wang Y., Deng J.L., Hu Y.C. (2021). *Ageratina adenophora* disrupts the intestinal structure and immune barrier integrity in rats. Toxins.

[28] Colucci R., Pellegrini C., Fornai M., Tirotta E., Antonioli L., Renzulli C., Ghelardi E., Piccoli E., Gentile D., Benvenuti L. (2018). Pathophysiology of NSAID-associated intestinal lesions in the rat: Luminal bacteria and mucosal inflammation as targets for prevention. Front. Pharmacol..

[29] Tang M., Yuan D., Liao P. (2021). Berberine improves intestinal barrier function and reduces inflammation, immunosuppression, and oxidative stress by regulating the NF-kappaB/MAPK signaling pathway in deoxynivalenol-challenged piglets. Environ. Pollut..

[30] Maloy K.J., Powrie F. (2011). Intestinal homeostasis and its breakdown in inflammatory bowel disease. Nature.

[31] Deng X., Wang Y., Tian L., Yang M., He S., Liu Y., Khan A., Li Y., Cao J., Cheng G. (2021). *Anneslea fragrans* Wall. ameliorates ulcerative colitis via inhibiting NF-kappaB and MAPK activation and mediating intestinal barrier integrity. J. Ethnopharmacol..

[32] Zhong G., Wan F., Lan J., Jiang X., Wu S., Pan J., Tang Z., Hu L. (2021). Arsenic exposure induces intestinal barrier damage and consequent activation of gut-liver axis leading to inflammation and pyroptosis of liver in ducks. Sci. Total Environ..

[33] Zhao L., Geng T., Sun K., Su S., Zhao Y., Bao N., Pan L., Sun H., Li M. (2020). Proteomic analysis reveals the molecular mechanism of *Hippophae rhamnoides* polysaccharide intervention in LPS-induced inflammation of IPEC-J2 cells in piglets. Int. J. Biol. Macromol..

[34] Zhuang S., Zhong J., Zhou Q., Zhong Y., Liu P., Liu Z.J. (2019). Rhein protects against barrier disruption and inhibits inflammation in intestinal epithelial cells. Int. Immunopharmacol..

[35] Zmora N., Levy M., Pevsner-Fischer M., Elinav E. (2017). Inflammasomes and intestinal inflammation. Mucosal Immunol..

[36] Ge L., Lin Z., Le G., Hou L., Mao X., Liu S., Liu D., Gan F., Huang K. (2020). Nontoxic-dose deoxynivalenol aggravates lipopolysaccharides-induced inflammation and tight junction disorder in IPEC-J2 cells through activation of NF-kappaB and LC3B. Food Chem. Toxicol..

[37] Zong S., Yang L., Park H.J., Li J. (2020). Dietary intake of *Lycium ruthenicum Murray* ethanol extract inhibits colonic inflammation in dextran sulfate sodium-induced murine experimental colitis. Food Funct..

[38] Kebaier C., Chamberland R.R., Allen I.C., Gao X., Broglie P.M., Hall J.D., Jania C., Doerschuk C.M., Tilley S.L., Duncan J.A. (2012). *Staphylococcus aureus* alpha-hemolysin mediates virulence in a murine model of severe pneumonia through activation of the NLRP3 inflammasome. J. Infect. Dis..

[39] Peng L.C., Jiang J.L., Chen T.T., Xu D.Y., Hou F.Q., Huang Q.Y., Peng Y.Y., Ye C., Hu D.L., Fang R.D. (2021). Toxic Shock Syndrome Toxin 1 Induces Immune Response via the Activation of NLRP3 Inflammasome. Toxins.

[40] Liao P., Li Y.H., Li M.J., Chen X.F., Yuan D.X., Tang M., Xu K. (2020). Baicalin alleviates deoxynivalenol-induced intestinal inflammation and oxidative stress damage by inhibiting NF-kappa B and increasing mTOR signaling pathways in piglets. Food Chem. Toxicol..

[41] Shen J., Cheng J.Z., Zhu S.G., Zhao J., Ye Q.Y., Xu Y.Y., Dong H.L., Zheng X.H. (2019). Regulating effect of baicalin on IKK/IKB/NF-kB signaling pathway and apoptosis-related proteins in rats with ulcerative colitis. Int. Immunopharmacol..

[42] Wan P., Xie M.H., Chen G.J., Dai Z.Q., Hu B., Zeng X., Sun Y. (2019). Anti-inflammatory effects of dicaffeoylquinic acids from *Ilex kudingcha* on lipopolysaccharide-treated RAW264.7 macrophages and potential mechanisms. Food Chem. Toxicol..

[43] Song S.Y., Chi D.H., Bae C.H., Kim Y.D. (2014). Staphylococcus enterotoxin A induces MUC5B expression via Toll-like receptor 2, extracellular signal-regulated kinase 1/2, and p38 mitogen-activated protein kinase in human airway epithelial cells. Am. J. Rhinol. Allergy.

[44] Shao D.Z., Lee J.J., Huang W.T., Liao J.F., Lin M.T. (2004). Inhibition of nuclear factor-kappa B prevents staphylococcal enterotoxin A-induced fever. Mol. Cell. Biochem..

[45] Mudili V., Makam S.S., Sundararaj N., Siddaiah C., Gupta V.K., Rao P.V.L. (2015). A novel IgY-Aptamer hybrid system for cost-effective detection of SEB and its evaluation on food and clinical samples. Sci. Rep..

[46] Li H.N., Yuan F., Luo Y.J., Wang J.F., Zhang C.B., Zhou W.E., Ren Z.Q., Wu W.J., Zhang F. (2017). Biosynthesis of staphylococcal enterotoxin A by genetic engineering technology and determination of staphylococcal enterotoxin A in water by HPLC-ESI-TOF. Environ. Sci. Pollut. Res. Int..

[47] Zhang H., Yan A., Liu X., Ma Y., Zhao F., Wang M., Loor J.J., Wang H. (2021). Melatonin ameliorates ochratoxin A induced liver inflammation, oxidative stress and mitophagy in mice involving in intestinal microbiota and restoring the intestinal barrier function. J. Hazard. Mater..

