# Supplementary material for: Staphylococcal Enterotoxin A Induces Intestinal Barrier Dysfunction and Activates NLRP3 Inflammasome via NF-κB/MAPK Signaling Pathways in Mice
Source: Toxins (Basel). 2022 Jan 1;14(1):29. doi: 10.3390/toxins14010029 (PMC8779132; doi:10.3390/toxins14010029)
Supplement: Supplementary file 1 [file toxins-14-00029-s001.zip › Supplementary Materials.pdf]

Supplementary Materials: Staphylococcal enterotoxin A induces intestinal barrier dysfunction and activates NLRP3 inflammasome via NF-κB/MAPK signaling pathways in mice

Chunmei Liu, Kunmei Chi, Meng Yang and Na Guo*

Table S1. The primary antibodies used in this study.

Antibody	Company	Catalog number	Dilutions of Western blot
Staphylococcal Enterotoxin A (SEA)	Sigma-Aldrich (St Louis, MO, USA)	S7656	1:20000
ZO-1	Affinity Biosciences (Jiangsu, China)	AF5145	1:1000
Occludin	Proteintech (Wuhan, China)	27260-1-AP	1:4000
NLRP3	Affinity Biosciences	DF7438	1:1000
Caspase-1/P20/P10	Proteintech	22915-1-AP	1:1000
ASC	Cell Signaling Technology (Boston, USA)	13833S	1:1000
ASC	Santa Cruz (Dallas, Texas, USA)	sc-22514-R	1:200
Cleaved-IL-1β	Affinity Biosciences	AF4006	1:1000
p38 MAPK (D13E1)	Cell Signaling Technology	8690T	1:1000
Phospho-p38 MAPK (Thr180/Tyr182) (D3F9)	Cell Signaling Technology	4511T	1:1000
p44/42 MAPK (Erk1/2) (137F5)	Cell Signaling Technology	4695T	1:1000
Phospho-p44/42 MAPK (Erk1/2) (Thr202/Tyr204) (D13.14.4E)	Cell Signaling Technology	4370T	1:1000
JNK	Cell Signaling Technology	9252T	1:1000
Phospho-SAPK/JNK (Thr183/Tyr185) (81E11)	Cell Signaling Technology	4668T	1:1000
NF-κB P65	Proteintech	10745-1-AP	1:1000
Anti-phospho-NF-kappaB p65 (pSer311)	Sigma-Aldrich (St Louis, MO, USA)	SAB4504482	1:1000
Lamin B1	Beyotime (Shanghai, China)	AF1408	1:1000
GAPDH	Proteintech	60004-1-Ig	1:20000

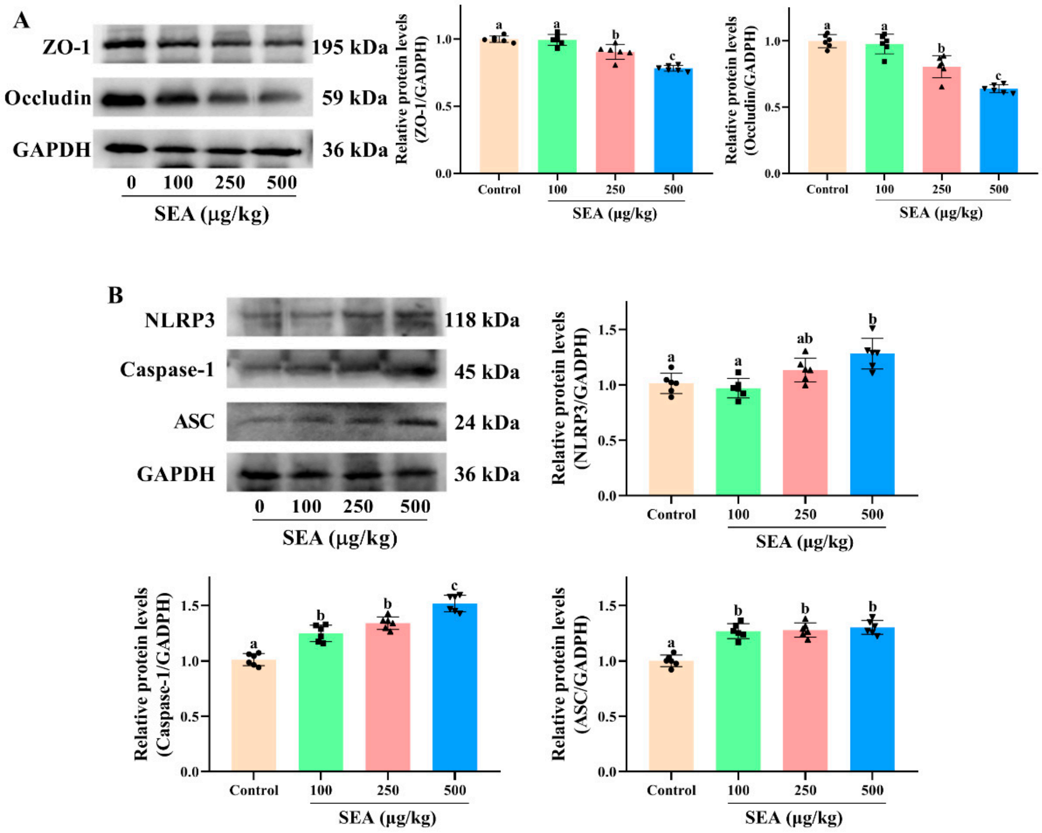

Figure S1. Western blot results of duodenum tissues of SEA-exposed mice. (A) Relative protein expression of ZO-1 and Occludin. (B) Relative protein expression of NLRP3, Caspase-1 and ASC. All data were expressed as mean ± SD (n = 6). Different lowercase letters showed significant difference between different groups, $p < 0.05$.

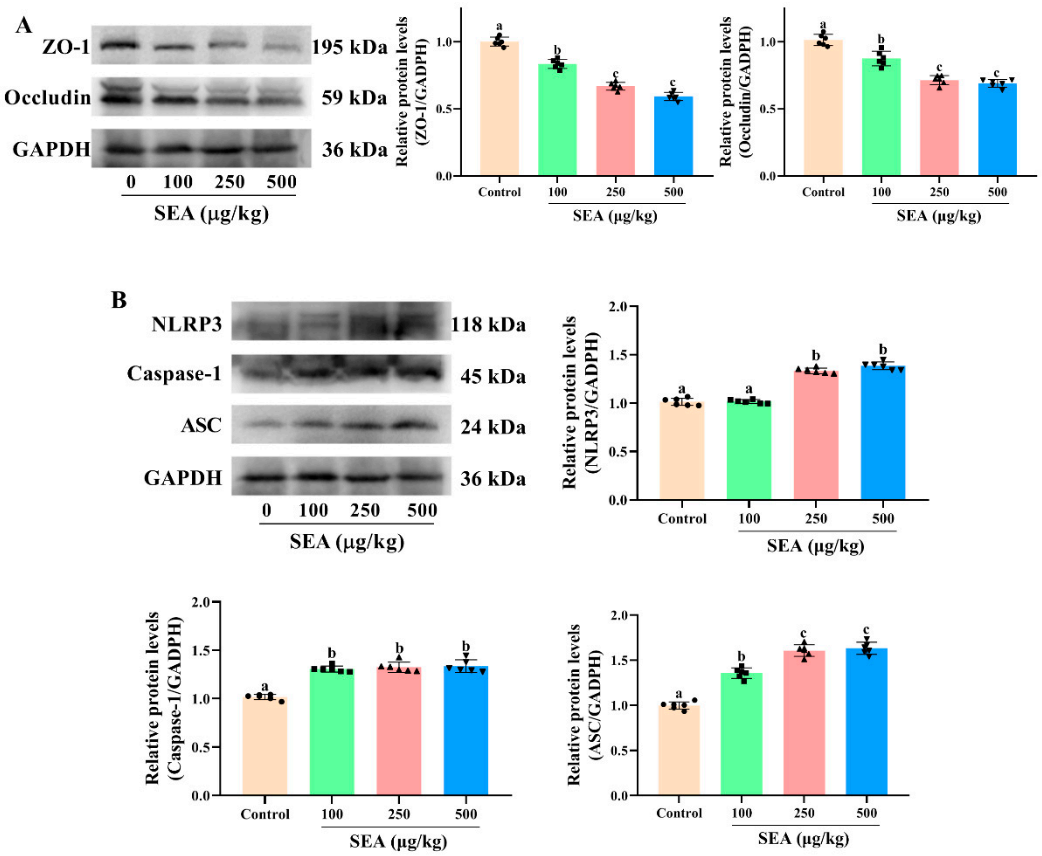

Figure S2. Western blot results of ileum tissues of SEA-exposed mice. (A) Relative protein expression of ZO-1 and Occludin. (B) Relative protein expression of NLRP3, Caspase-1 and ASC. All data were expressed as mean ± SD (n = 6). Different lowercase letters showed significant difference between different groups, $p < 0.05$.
